# Surviving a Dry Future: Abscisic Acid (ABA)-Mediated Plant Mechanisms for Conserving Water under Low Humidity

**DOI:** 10.3390/plants6040054

**Published:** 2017-11-04

**Authors:** Frances C. Sussmilch, Scott A. M. McAdam

**Affiliations:** 1 School of Biological Sciences, University of Tasmania, Hobart TAS 7001, Australia; frances.sussmilch@uni-wuerzburg.de; 2 Institute for Molecular Plant Physiology and Biophysics, University of Würzburg, D-97082 Würzburg, Germany; 3 Purdue Center for Plant Biology, Department of Botany and Plant Pathology, Purdue University, West Lafayette, IN 47907, USA

**Keywords:** stomata, humidity, vapor pressure deficit (VPD), abscisic acid (ABA), 9-*cis*-epoxycarotenoid dioxygenase (NCED), water deficit stress, evolution, sensing water status

## Abstract

Angiosperms are able to respond rapidly to the first sign of dry conditions, a decrease in air humidity, more accurately described as an increase in the vapor pressure deficit between the leaf and the atmosphere (VPD), by abscisic acid (ABA)-mediated stomatal closure. The genes underlying this response offer valuable candidates for targeted selection of crop varieties with improved drought tolerance, a critical goal for current plant breeding programs, to maximize crop production in drier and increasingly marginalized environments, and meet the demands of a growing population in the face of a changing climate. Here, we review current understanding of the genetic mechanisms underpinning ABA-mediated stomatal closure, a key means for conserving water under dry conditions, examine how these mechanisms evolved, and discuss what remains to be investigated.

## 1. Introduction

Water availability is a major limiting factor for plant survival and growth, and is one of the most significant constraining factors for crop production. Water scarcity is already a global issue, with 71% of the world’s population currently experiencing periods of moderate to severe water scarcity [1]. Climate change will continue to exacerbate issues with water availability through increased temperatures and the frequency and/or severity of droughts [2,3,4]. Water resources will be placed under further pressure in the future by the increased demands stemming from population growth, as the world population climbs to reach up to 12.3 billion people by 2100 [5]. To meet the immense challenge of feeding this growing population in the face of a changing climate, it will be necessary to develop crop varieties that can survive in drier and increasingly marginalized environments. Achieving this goal will require a detailed understanding of the mechanisms that can enable plants to survive in dry environments [6].

Stomata, the pores which allow gas exchange in photosynthetic tissues protected by a cuticle, are the largest point of water loss from a plant, as water evaporates from the humid sub-stomatal cavity within the plant into the dry atmosphere [7]. Mechanisms have evolved for regulating the aperture of the stomatal pore, mediated by the hormone abscisic acid (ABA) [8,9], that enable the need for photosynthetic gas exchange to be balanced with minimizing water loss under dry conditions [10]. These mechanisms facilitate the rapid, precise and flexible control of stomatal aperture, and enable it to be decoupled from leaf water content, yielding a competitive advantage in dry or changing environments. ABA-mediated stomatal control enables a wide variety of behaviors, ranging from keeping stomata open longer for increased photosynthesis under dry conditions (in combination with desiccation tolerance mechanisms) [11], to circadian clock regulation of ABA levels (enabling anticipation of regular diurnal fluctuations in environmental conditions) [12,13]. Numerous genes that are involved in ABA biosynthesis or signaling, or downstream targets of the ABA-signaling pathway, have been identified as candidates for targeted breeding of crop varieties with improved drought tolerance [14,15,16,17,18,19].

Atmospheric humidity is one of the most dynamic and fast-changing environmental conditions that influences leaf water status throughout the day. Air humidity, or, more precisely, the vapor pressure difference between the leaf and the atmosphere (VPD), is a major factor affecting terrestrial net primary production [20]. In this review, we summarize our current understanding of the angiosperm mechanisms for ABA-mediated closure in response to low humidity and explore how these mechanisms evolved.

## 2. ABA-Mediated Humidity Responses in Angiosperms

A significant decrease in humidity/increase in VPD triggers a rapid rise in ABA levels and ABA-induced stomatal closure in diverse angiosperm species [21,22,23,24,25]. Rather than directly sensing changes in humidity, it has been proposed that angiosperms detect rapid changes in VPD , sustained water deficit stress, and osmotic stressby sensing alterations in plant water status via cell turgor, a reduction in cell volume or the relationship between the cell membrane and cell wall [26,27,28,29]. Accordingly, manipulating external pressure, which likely alters the cell membrane–wall interactions via a reduction in cell turgor or relative water content, induces the same ABA-biosynthetic response as a VPD transition [26,27,30]. ABA plays a major role in regulating stomatal responses to VPD in angiosperms, as evidenced by the wilty phenotypes of ABA biosynthetic and signaling mutants at high VPD, combined with significantly impaired stomatal responses to increased VPD in sextuplet ABA-receptor mutants and mutants in the key ABA signaling gene *OPEN STOMATA1* (*OST1*) [31,32]. The speed of the stomatal response to VPD has previously led to the suggestion that ABA levels rise rapidly due to the release of fettered ABA [33], by the single-step hydrolyzation of stored, conjugated ABA–glucose ester (ABA–GE) [34,35]. While this hydrolysis pathway appears important for plants to respond to sustained dehydration stress [35], it does not appear to play a significant role in fast VPD responses across diverse angiosperm species, as ABA–GE levels do not change sufficiently, or even in the right direction (i.e., decrease) in some species, to account for the rapid increase in ABA levels under transient (20 min) VPD transitions [21]. Recent evidence suggests, however, that ABA–GE may play a role in longer-term VPD transitions (over the course of several hours from morning to afternoon) in a perennial plant species [36].

Local ABA levels can also increase through a decreased rate of catabolism, with the first and key step of the oxidative pathway for ABA catabolism catalyzed by ABA 8′-hydroxylases [37]. However, the expression of the *CYP707* family genes, which encode these enzymes, are in fact upregulated (rather than downregulated) by water deficit stress or altering external pressure, likely via a pathway that is at least partially ABA-dependent, increasing the rate of ABA catabolism under water deficit stress [30,37,38,39]. Thus, regulation of catabolism does not account for the rapid increases in ABA levels induced by high VPD, either.

Instead, increased VPD (or altering external pressure) has been found to trigger rapid de novo biosynthesis of ABA in the leaf, by upregulation of at least one key gene that encodes a 9-*cis*-epoxycarotenoid dioxygenase (NCED) enzyme [21,30], the rate-limiting enzyme in the ABA biosynthesis pathway [40,41]. NCED enzymes catalyze the first committed step in ABA biosynthesis in plants, the oxidative cleavage of the 9’-*cis*-epoxycarotenoids neoxanthin and violaxanthin (C40), to produce xanthoxin (C15) [42,43,44]. This pathway is most thoroughly characterized in the model angiosperm *Arabidopsis thaliana*, wherein *NCED3* is the key, rate-limiting gene expressed in leaves in response to water deficit stress [45], and the only gene within the ABA biosynthesis pathway to be significantly upregulated within the time-frame of the stomatal VPD response [21], which occurs within minutes [30].

Leaves, rather than roots, are the main site of ABA biosynthesis in the plant [46,47,48]. Within leaves, ABA may be synthesized in the guard cells themselves, where all genes within the ABA biosynthetic pathway are expressed [49], or in the vascular tissue, where expression of a number of biosynthetic genes (including *NCED3*) is highest [50,51,52], with subsequent transport into guard cells occurring through either passive diffusion, or active transport by proteins including ATP-BINDING CASSETTE (ABC) transporters [53,54,55]. The results of a recent study indicate that ABA derived from the guard cells or the phloem companion cells is functionally equivalent in restoring VPD responses in an ABA-deficient mutant [32].

ABA is detected by receptors within the PYRABACTIN RESISTANCE 1 (PYR)/PYR1-LIKE (PYL)/REGULATORY COMPONENT OF ABA RECEPTOR (RCAR) family in the guard cells [56,57,58]. ABA-bound receptors, in turn, bind clade A protein phosphatase type 2C (PP2C) proteins, including ABA INSENSITIVE1 (ABI1), ABI2 and HOMOLOG OF ABI1/2 (HAB1), alleviating PP2C inhibition of the key ABA-signaling kinase, OST1 [59,60,61,62,63,64,65,66,67]. OST1 activates downstream targets, including S- and R-type anion channels in the guard cell membrane, causing a flow of anions from the guard cells [68,69,70,71]. This depolarizes the cell membrane, activating the potassium channel GATED OUTWARDLY-RECTIFYING K^+^ CHANNEL (GORK), resulting in a flow of cations and further reducing the osmotic potential of the guard cells, which deflate and close the stomatal pore [72,73]. An additional ABA-sensitive pathway involving calcium-dependent protein kinases (CPKs) also activates the S-type anion channels SLOW ANION CHANNEL 1 (SLAC1) and SLAC1 HOMOLOG 3 (SLAH3), and the potassium channel GORK, independently of OST1 [74,75,76,77,78,79]. Receptor binding of ABA also relieves the direct inhibition of these channels by PP2Cs [80,81,82]. Stomatal re-opening is further inhibited under water deficit stress, by the inhibition of the inward-rectifying potassium channel K^+^ CHANNEL IN ARABIDOPSIS THALIANA 1 (KAT1) by OST1, SLAC1, and SLAH3 [62,83,84].

## 3. Possible Candidates for the Angiosperm Pathway for Rapid VPD Responses

Despite the importance of the early stages of the angiosperm pathway for rapid responses to VPD, which links the sensing of altered cellular properties to transcriptional upregulation of the key *NCED* gene, this pathway remains uncharacterized, even in Arabidopsis. This pathway likely includes at least one (a) sensor that detects the cellular change caused by increased VPD (b) transcriptional regulator that upregulates transcription of the key *NCED* gene, and may also include one or more intermediate proteins that relay the signal between these. As multiple environmental signals, including increased VPD, sustained drought and osmotic stress (including the osmotic component of salt stress) converge upon *NCED* transcriptional upregulation for increased ABA biosynthesis [45,85,86,87,88], it is likely that members of the early VPD-response pathway are shared between these stress response pathways that detect and respond to changes in plant water status.

### 3.1. Candidates for the Plant Water Status Sensor that Triggers VPD Responses

A sensor that detects subtle cellular changes associated with increased VPD could be either a mechanosensor that detects mechanical changes in cell shape or volume, or an osmosensor that detects increased concentration of internal solutes as water is lost from the cell. Previously, the transmembrane protein Arabidopsis Histidine Kinase1 (AHK1), a homolog of the yeast osmosensor synthetic lethal of N-end rule1 (SLN1), was favored as a candidate for the unknown sensor in the VPD response pathway [89,90,91]. However, there is no significant difference in the induction of *NCED3* or other ABA biosynthetic genes, or in stomatal responses, between wild type and *ahk1* null mutant plants in response to the application of external pressure on the leaf simulating a natural increase in VPD, within the time-frame for rapid VPD responses, indicating that *AHK1* does not play a critical role in this pathway [30]. A role for *AHK1* in the regulation of ABA biosynthesis under sustained water deficit stress has similarly been discounted [92].

Some studies have found that protoplasts do not synthesize ABA in response to dehydration stresses [93,94], suggesting that the presence of a cell wall is important for sensing associated changes in cell properties. Accordingly, proteins linked to sensing the integrity of the cell wall, or the relationship between the cell wall and plasma membrane, have also been highlighted as possible candidates for a role in sensing and signaling for dehydration stress responses [95,96,97,98]. In animals, Arg–Gly–Asp (RGD) tripeptide motifs are conserved in extracellular matrix proteins, and membrane-bound integrin proteins that bind to these RGD motifs are important for transducing signals between the environment and the cell interior [99,100,101,102]. Plant RGD-binding proteins are not only important for interactions between the cell wall and plasma membrane [103,104], they also play an important role in triggering ABA biosynthesis in response to osmotic stress, as artificial RGD-containing peptides have been found to block this process through competitive binding [105,106]. Although plants lack clear orthologs to mammalian RGD-binding integrins [107], a number of plant proteins that are capable of binding to RGD tripeptides have been identified, including integrin-like proteins [108,109,110], and some members of the large receptor-like kinase (RLK) family [111]. Some other, non-RGD binding members of the RLK family have also been linked to a role in sensing cell wall integrity, including wall associated kinases (WAKs) that bind pectins in the cell wall [112], and *Catharanthus roseus* RLK1-like proteins (CrRLK1Ls) and lectin receptor kinases (LecRKs), which have extracellular domains thought to bind carbohydrates from the intact cell wall or derived from degraded cell wall components [113,114,115]. Some plasma membrane proteins, including RLKs, a START domain protein (with a putative role in lipid binding), and an aspartic protease, have been found to alter plant tolerance to drought or hyperosmotic stress, expression of ABA biosynthesis genes (including *NCED* genes), and/or ABA levels [98,116,117,118,119,120,121,122,123]. However, there is a positive feedback loop by which ABA upregulates its own biosynthesis pathway, by upregulating expression of *NCED* genes [49,50,85,124], so it will require additional experimentation to separate any role these genes may play upstream of ABA biosynthesis for initial responses to increased VPD, from downstream roles in ABA-signaling that feed back into ABA biosynthesis. Nonetheless, a number of these proteins have yet to be functionally characterized, and remain possible candidates for the unknown plant sensor involved in VPD responses.

Calcium signaling has also been suggested to be involved in inducing ABA biosynthesis in response to a range of environmental stresses including drought, salt, and low temperature [95,97,125]. Although testing the role of calcium signaling in stress responses is also complicated by the role of Ca^2+^ as a second messenger in downstream ABA-signaling [126,127], calcium fluxes offer a means for fast signaling, with dramatic increases in cytosolic Ca^2+^ concentration recorded within seconds of hyperosmotic or salt treatments [128,129]. A number of families of Ca^2+^-permeable mechanosensitive or osmosensitive ion channels have been identified. The stretch-activated MID1-COMPLEMENTING ACTIVITY (MCA) proteins are capable of sensing increases in cell turgor due to hypo-osmotic stress [130,131,132]. Similarly, some MscS-like (MSL) proteins have a role in protection from hypo-osmotic stresses [133], with some similarity to bacterial homologs [134], but the roles of the plant MSL family appear diverse and remain to be fully characterized [135]. The hyper-osmolality-gated calcium-permeable channel REDUCED HYPEROSMOLARITY-INDUCED [Ca^2+^]_i_ INCREASE (OSCA)1 is necessary for stomatal closure in response to osmotic stress, and is thought to act upstream of ABA biosynthesis, as ABA responses are normal in the *osca1* mutant [136]. However, quantification of either ABA levels or *NCED* transcript levels has not been reported for this mutant, so this remains to be confirmed. OSCA1 and related proteins contain a conserved DUF221 domain that functions as an osmotic-sensing calcium channel [137], and other DUF221 proteins have also been characterized as having a role in early hyperosmotic or drought stress responses [138,139].

### 3.2. Candidate Transcriptional Regulators for Key NCED Genes during VPD Responses

Although the specific transcription factor/s that upregulate *NCED* gene expression in response to increased VPD have not yet been characterized, a number of candidates have been identified. Firstly, *ATAF1*, a member of the plant-specific NAC transcription factor family, directly upregulates *NCED3* expression in Arabidopsis [140,141], and is itself upregulated within the first 30 min of drought treatment [142]. However, *ATAF1* is also induced by ABA [143], and *ataf1* mutants show similar ABA levels to wild-type plants [141], so it is not yet clear whether *ATAF1* acts during initial induction of ABA biosynthesis, or is limited to a role in downstream ABA-signaling.

Secondly, a number of genes from the WRKY transcription factor family, one of the largest families of transcriptional regulators in plants, have also been identified as regulating *NCED* genes. In Arabidopsis, WRKY57 binds to the promoter of *NCED3* and directly stimulates its transcription to induce ABA biosynthesis [144]. In banana, four transcription factors from diverse clades within the WRKY family, MaWRKY31, MaWRKY33, MaWRKY60, and MaWRKY71, were also found to directly bind to the promoter sequences of banana *NCED* homologs [145]. This suggests that there may be a number of WRKY transcription factors that can regulate the transcription of *NCED* genes, either redundantly or in response to different signals.

Lastly, the Arabidopsis trithorax-like factor ARABIDOPSIS HOMOLOG OF TRITHORAX1 (ATX1) alters *NCED3* chromatin by the trimethylation of histone H3 at lysine 4 (H3K4me3). As a result, binding of RNA polymerase II and subsequent transcription is enhanced in response to dehydration stress [146]. The loss of *ATX1* function results in decreased tolerance to dehydration stress due to decreased *NCED3* transcription, decreased ABA levels, and increased rates of transpiration through stomata with larger apertures [146]. *ATX1* has not yet been tested for a role in VPD responses, but remains a potential candidate.

## 4. The Evolution of ABA-Mediated Plant Humidity Responses

**Figure 1:**
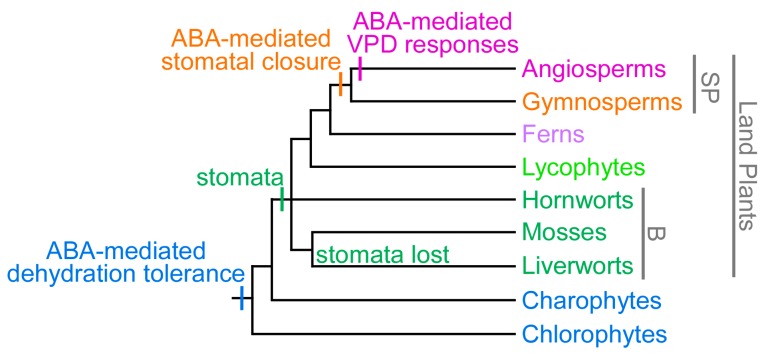
The gradualistic model for the evolution of abscisic acid (ABA)-mediated control of stomatal aperture. The relative timing of appearance of key traits is indicated on the current, most parsimonious phylogeny for land plants (not drawn to scale) that recognizes current uncertainty in the relationships between bryophytes (B) and vascular plants [161]. A role for ABA in dehydration/desiccation tolerance mechanisms is observed in the green algal groups, the chlorophytes and the charophytes, predating the evolution of stomata in a land plant ancestor. Stomatal closure in response to biologically relevant levels of ABA are restricted to the seed plants (SP) [149,152]. ABA-mediated responses to humidity/vapor pressure deficit (VPD) evolved in an angiosperm ancestor, after divergence of the gymnosperm lineage [24,26]. The hypothesis of a monophyletic origin of stomata is adopted for simplicity [7].

Although the timing of the evolution of ABA-mediated stomatal responses in land plants is the topic of current debate [147,148], the results of physiological studies provide strong evidence that ABA-mediated stomatal closure in response to high VPD is a trait unique to angiosperms [24,31,149], according to the gradualistic model for evolution of ABA-mediated stomatal responses (Figure 1). A critical requirement for an ABA-mediated stomatal response to VPD is the ability to rapidly upregulate ABA to sufficient levels within a suitable timeframe, in order to respond to rapid fluctuations in VPD. While gymnosperms respond to ABA by closing their stomata [149,150,151,152], similar to angiosperms (Figure 1), the rate of ABA-biosynthesis is significantly slower in gymnosperms, with angiosperms capable of increasing ABA to levels sufficient to induce stomatal closure within minutes [30,153], while gymnosperms require hours [149]. This difference in speed is likely due at least in part to the presence of a dedicated ABA-specific short-chain dehydrogenase/reductase (SDR) enzyme in angiosperms, ABA DEFICIENT 2 (ABA2) [50,154], which is not represented in other plant lineages [23,147,155]. The leaky nature of angiosperm *aba2* mutants, which can slowly synthesize a small amount of ABA, indicate that other, non-specific SDRs are capable of catalyzing this step, but are less efficient [23,154,156,157]. In addition, it is possible that the rate of upregulation of key, rate-limiting *NCED* genes may be faster in angiosperms than older plant lineages, but this remains to be determined. In response to either a transition to high VPD or application of corresponding external pressure to the leaf, foliar ABA levels do not rise sufficiently to trigger stomatal closure in ferns or gymnosperms [24,26]. In contrast to the predominantly “active”, ABA-mediated angiosperm stomatal responses to VPD, gymnosperms, ferns and lycophytes show highly predictable, “passive” control of stomatal aperture in response to VPD transitions, resulting solely from reduced guard cell water content and turgor under conditions of increased transpiration [24,149,158]. This suggests that key elements of the pathway for rapid ABA-biosynthesis in response to increased VPD, evolved or acquired this function in an angiosperm ancestor, after divergence of the gymnosperm lineage (Figure 1). Ancestral passive mechanisms for stomatal closure in response to VPD transitions may also play a role in angiosperm stomatal responses to VPD, in addition to active, ABA-mediated mechanisms, however the extent to which this ancestral response contributes to stomatal behavior in these species is currently debated [24,32,159,160].

In contrast to seed plants, the stomata of plants from basal vascular plant lineages, including lycophytes and ferns, do not close in response to ABA when it is applied at biologically relevant concentrations (i.e., corresponding to levels found endogenously) [149,152]. Extremely high concentrations of ABA, more than 1000× higher than endogenous levels, can elicit a reduction in stomatal aperture in some moss [162], hornwort [163], lycophyte [164], and fern species [165,166]. However, as these levels are not found endogenously, their biological relevance is debatable, and even these extremely high levels elicit only minor responses in basal land plants, which contrasts sharply with the complete stomatal closure induced by considerably lower, biologically relevant ABA levels in seed plants [152,167,168]. These findings indicate that one or more of the key mechanisms required for ABA-mediated stomatal closure evolved in a seed plant ancestor, after divergence from lycophyte and fern lineages. Indeed, a functional homolog pair for two key proteins involved in ABA-mediated stomatal closure in angiosperms, OST1 and SLAC1 [68,69,169,170], was found to be absent in the model lycophyte *Selaginella moellendorffii*, and all fern OST1–SLAC pairs tested thus far have been found to be similarly non-functional [171]. While a functional OST1–SLAC pair has been identified in the moss *Physcomitrella patens* [172], this pair does not appear to show the guard cell specificity required for ABA-mediated stomatal responses [162,173]. Comparisons between stomatal behavior between bryophytes and vascular plants are further complicated by differences in the role of stomata between these plant lineages. While stomata function in gas exchange and close to minimize plant dehydration under dry conditions in vascular plants, bryophyte stomata have an apparently ancient role in facilitating the desiccation of spore capsules, and once mature, stomatal structure and behavior indicates that these stomata open once, and never close [174,175,176,177,178].

**Figure 2:**
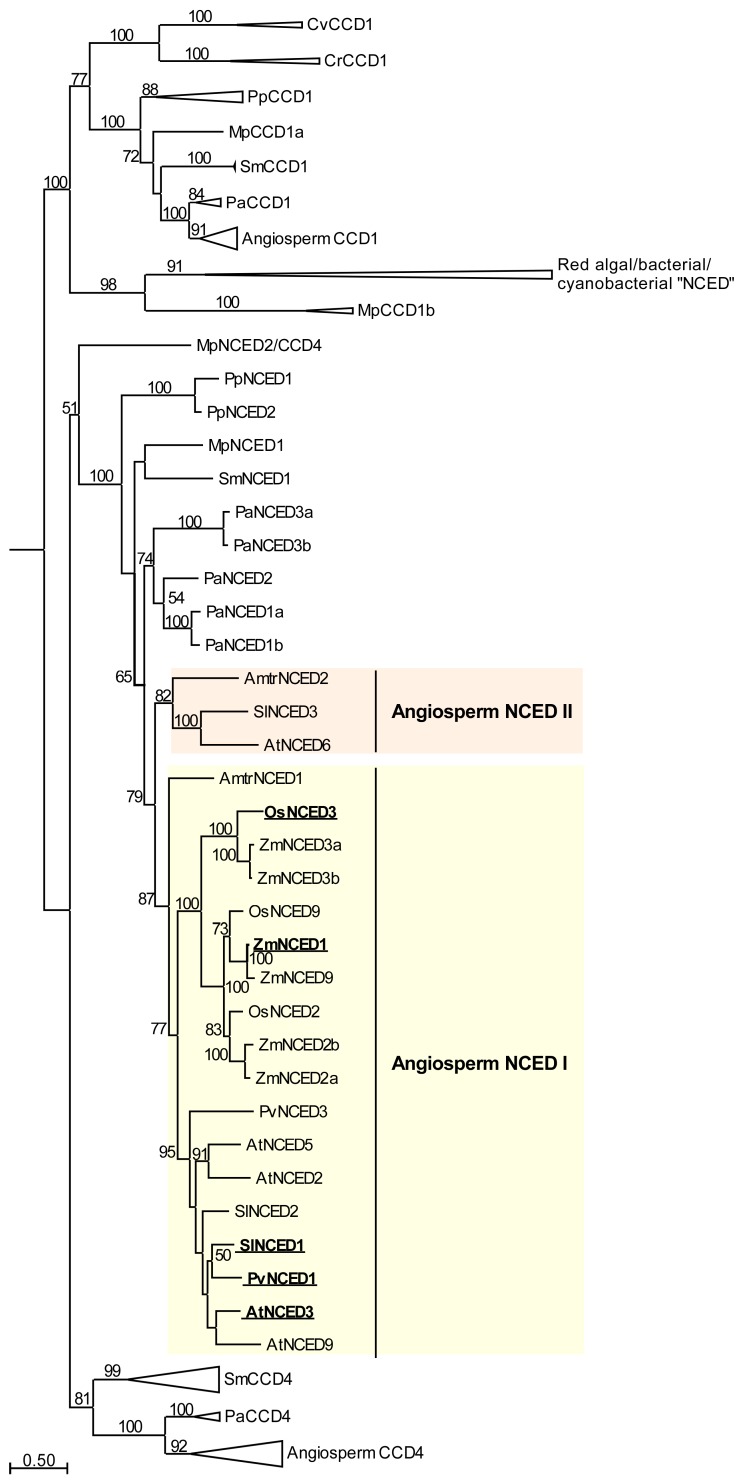
*NCED* and related *CCD4* and *CCD1* gene subfamilies from land plants and green algae, including genes previously called *NCED* genes from red algae, bacteria, and cyanobacteria. The maximum likelihood phylogenetic was generated using PhyML 3.0 with SmartModel Selection [226] from a MAFFT alignment of full length predicted protein sequences for genes identified by reciprocal BLASTp searches, initially using AtNCED3 protein as a query sequence, in available resources for representative angiosperm (Amtr, *Amborella trichopoda*; At, *Arabidopsis thaliana*; Os, *Oryza sativa*; Pv, *Phaseolus vulgaris*; Sl, *Solanum lycopersicum*; Zm, *Zea mays*), gymnosperm (Pa, *Picea abies*), lycophyte (Sm, *Selaginella moellendorffi*), moss (Pp, *Physcomitrella patens*), liverwort (Mp, *Marchantia polymorpha*), and green algal (Cr, *Chlamydomonas reinhardtii*; Cv, *Chlorella variabilis*) species. The top BLASTp hits for AtNCED3 in green algal genomes belong to *CrCCD1* and *CvCCD1* subclades. Genes previously called “*NCED*” genes in red algal, bacterial, and cyanobacterial species [194,195] are also included. Genes previously found to be strongly upregulated in leaves under dehydration stress are shown in bold and underlined [41,45,86,198,199]. Angiosperm *NCED* subclades are labelled. Some *CCD1* and *CCD4* subclades have been collapsed for figure clarity. Details of sequences and collapsed clades are given in Appendix A. Bootstrap values from 1000 replicates are shown as percentages for clades with >50% support. The scale bar indicates amino acid changes.

While together these findings indicate that ABA-mediated stomatal responses evolved relatively recently in a seed plant ancestor (Figure 1), ABA and key proteins involved in ABA biosynthesis, perception and signaling, including NCED enzymes (Figure 2), PYR/PYL/RCAR receptors [179,180], inhibitory PP2C phosphatases [179,181], and OST1-type SNF1-RELATED KINASE 2 (SnRK2) family proteins [171,172,182], have ancient origins, and can be found across land plants spanning from bryophytes (including liverworts, which lack stomata) to angiosperms. ABA is not limited to plants, but is found in a wide variety of organisms including bacteria, fungi, and animals [182,183,184]. Even in the most basal aquatic plants, green algae, ABA is upregulated in response to stresses, including drought, osmotic, salt, pH, high light and heat stresses, and nitrogen deficiency [185,186,187,188,189,190]. While algae, bacteria, cyanobacteria, and fungi can synthesize ABA, they do not possess *NCED* genes, which are found only in land plants (Figure 2), and instead, these organisms are thought to synthesize ABA directly via the C15 compound farnesyl diphosphate (FDP), derived from the mevalonic acid (MVA) pathway [182,191,192,193]. Some genes from algae, bacteria, and cyanobacteria have previously been named as *NCED* genes [194,195,196], but these genes are, instead, members of the related *CAROTENOID CLEAVAGE DIOXYGENASE 1* (*CCD1*) subfamily within the larger *CCD* gene family (Figure 2).

The results of phylogenetic analysis of land plant *NCED* genes reveal two angiosperm subclades: I (containing Arabidopsis *NCED2*, *NCED3*, *NCED5*, and *NCED9* genes, and all Poaceae *NCED* genes) and II (including *AtNCED6*). Angiosperm *NCED* subclade I genes radiated separately in monocot and dicot ancestors, after divergence of these two lineages. All dicot genes with a key role in water deficit stress-induced ABA biosynthesis in leaves, *AtNCED3* [45], *PvNCED1* [41], and *SlNCED1* [86,197], are included within a single group in *NCED* subclade I. In contrast, genes from different groups within the monocot *NCED* genes, *OsNCED3* and *ZmNCED1*/*VP14*, are both strongly upregulated in leaves during water deficit stress [198,199], suggesting that there may be some diversity in the *NCED* genes fulfilling a role in water deficit stress-induced ABA biosynthesis in monocot leaves. Angiosperm *NCED* subclade II is represented in the basal angiosperm *Amborella trichopoda*, the rosid Arabidopsis and the asterid *Solanum lycopersicum*, but was not represented in the Poaceae species included in this analysis, or in the rosid *Phaseolus vulgaris* (Figure 2), suggesting that this group of *NCED* genes may have been lost at multiple points during angiosperm evolution.

The roles of ABA have evolved and changed through time, as evidenced by the diverse roles ABA plays in extant organisms. In algae, ABA affects nitrogen uptake, ATP levels, sugar metabolism, growth morphology, dormancy and desiccation tolerance [186,200,201,202,203]. In bryophytes, an ancient role for ABA in desiccation tolerance is also evident, via upregulation of proteins with a role in osmoregulation/osmoprotection to protect cells from desiccation-induced damage, including aquaporins, sugar transporters, metabolic enzymes, and late embryogenesis abundant (LEA) proteins, such as dehydrins [204,205,206,207,208,209]. A role for ABA in dehydration/desiccation tolerance is also evident in all other plant lineages [210,211,212,213,214], distinct from the role of ABA in desiccation prevention via stomatal closure in seed plants [8,152,215]. ABA has also been found to control a range of plant developmental processes, including spore/seed dormancy/germination, sex determination, leaf morphology, and plant growth [171,173,216,217,218,219,220,221,222,223,224,225]. Despite these diverse roles, there is overlap in the genetic pathways involved in ABA biosynthesis and signaling, and it is possible that the angiosperm VPD response pathway, responsible for rapid induction of key *NCED* genes in response to increased VPD, was co-opted from an older, existing pathway for dehydration-induced ABA biosynthesis; this possibility remains to be investigated.

## 5. Conclusions

The phytohormone ABA has evolved to control a variety of processes in plants, ranging from ancient roles in dehydration/desiccation tolerance and spore dormancy, seen in mosses, to desiccation prevention via ABA-mediated stomatal closure in seed plants. In angiosperms, the evolution of mechanisms for rapid ABA biosynthesis (within minutes), have enabled fast, ABA-mediated stomatal closure in response to changes in humidity/VPD. These mechanisms enable precise control of stomatal aperture, decoupled from leaf water content, yielding a competitive advantage in dry or changing environments. While significant progress has been made in the characterization of ABA biosynthesis enzymes and ABA perception and signaling pathways, the early VPD-response pathway, involved in initial regulation of the key rate limiting gene in the ABA biosynthesis pathway, *NCED*, remains unknown. Identification of this pathway will offer new targets for breeding crop varieties with improved drought tolerance, either through genetic engineering or more traditional, marker-assisted selection approaches, critical for feeding a growing population under a future, drier climate.

## Appendix A

Sequences were obtained from Phytozome [227], GenBank [228], or ConGenIE [229], as indicated.

**Table A1:** Details of Sequences Used in Phylogenetic Analysis.

Protein/Collapsed Subclade Name	Species	Accession	Source	Reference
Angiosperm CCD1	*Amborella trichopoda*	evm_27.model.AmTr_v1.0_scaffold00022.400 evm_27.model.AmTr_v1.0_scaffold00022.401	v1.0; Phytozome	This study
	*Arabidopsis thaliana*	AT3G63520 (AtCCD1)	TAIR10; Phytozome	[230]
	*Oryza sativa*	LOC_Os12g44310	v7; Phytozome	[231]
	*Phaseolus vulgaris*	Phvul.011G211200	v2.1; Phytozome	This study
	*Solanum lycopersicum*	Solyc01g087250 Solyc01g087260	iTAG2.4; Phytozome	[232]
	*Zea mays*	GRMZM2G057243	v1.0; Phytozome	[231]
Angiosperm CCD4	*Amborella trichopoda*	evm_27.model.AmTr_v1.0_scaffold00011.172	v1.0; Phytozome	This study
	*Arabidopsis thaliana*	AT4G19170 (AtCCD4)	TAIR10; Phytozome	[230]
	*Oryza sativa*	LOC_Os02g47510 LOC_Os12g24800	v7; Phytozome	[231]
	*Phaseolus vulgaris*	Phvul.002G120600	v2.1; Phytozome	This study
	*Solanum lycopersicum*	Solyc08g075480 Solyc08g075490	iTAG2.4; Phytozome	[232]
	*Zea mays*	GRMZM2G110192 GRMZM2G150363	Ensembl-18; Phytozome	[231]
AmtrNCED1	*Amborella trichopoda*	evm_27.model.AmTr_v1.0_scaffold00092.158	v1.0; Phytozome	[21]
AmtrNCED2		evm_27.model.AmTr_v1.0_scaffold00039.158		
AtNCED2	*Arabidopsis thaliana*	AT4G18350	TAIR10; Phytozome	[45,230]
AtNCED3		AT3G14440		
AtNCED5		AT1G30100		
AtNCED6		AT3G24220		
AtNCED9		AT1G78390		
CrCCD1	*Chlamydomonas reinhardtii*	Cre03.g149650 Cre08.g365825	v5.5; Phytozome	This study: top BLASTp hits for AtNCED3
CvCCD1 (prev. NCED)	*Chlorella variabilis*	EFN52762	GenBank	[196]
MpCCD1a	*Marchantia polymorpha*	Mapoly0003s0307	v3.1; Phytozome	This study
MpCCDb		Mapoly0012s0197 Mapoly0066s0017		
MpNCED1		Mapoly0015s0066		
MpNCED2/CCD4		Mapoly0149s0036		
OsNCED2	*Oryza sativa*	LOC_Os12g42280	v7; Phytozome	[231]
OsNCED3a		LOC_Os07g05940		
OsNCED9		LOC_Os03g44380		
PaCCD1	*Picea abies*	MA_10435932g0010 MA_210464g0010 MA_906445g0010	v1.0; ConGenIE	This study
PaCCD4		MA_10425950g0010 MA_90573g0010 MA_10428396g0010		
PaNCED1a		MA_10428505g0020		
PaNCED1b		MA_10428505g0010 comp88253_c0_seq1		
PaNCED2		MA_10434448g0010		
PaNCED3a		MA_10174788g0010		
PaNCED3b		MA_198304g0010		
PpCCD1	*Physcomitrella patens*	Pp3c12_22350V3.1 Pp3c18_17950V3.1 Pp3c21_12920V3.1 Pp3c22_6380V3.1	v3.3; Phytozome	[182,231]
PpNCED1		Pp3c16_17210V3.1		
PpNCED2		Pp3c25_4816V3.1		
PvNCED1	*Phaseolus vulgaris*	Phvul.005G051600	v2.1; Phytozome	[41]
PvNCED3		Phvul.007G198800		[21]
Red algal/bacterial/cyanobacterial “NCED”	*Cyanidioschyzon merolae*	XP_005538977 (CMS362C)	GenBank	[194,195]
	*Phaeodactylum tricornutum*	XP_002177588		
	*Trichodesmium erythraeum*	WP_011612676 (Tery_3212)		
SlNCED1 (NOT)	*Solanum lycopersicum*	Solyc07g056570	iTAG2.4; Phytozome	[197,232]
SlNCED2		Solyc08g016720		
SlNCED3		Solyc05g053530		
SmCCD1	*Selaginella moellendorffii*	165469 272067	v1.0; Phytozome	[155,231]
SmCCD4 (prev. SmNCEDa + c)	*Selaginella moellendorffii*	11287 11289 11292 11304 75383 79628 80651 91815 94523	v1.0; Phytozome	[155,231]
SmNCED1 (prev. SmNCEDb)		233638		
ZmNCED1 (VVP1)	*Zea mays*	GRMZM2G014392	Ensembl-18; Phytozome	This study; [199,231]
ZmNCED2a		GRMZM5G858784		
ZmNCED2b		GRMZM2G407181		
ZmNCED3a		GRMZM2G417954		
ZmNCED3b		GRMZM2G408158		
ZmNCED9		GRMZM5G838285		
